# Release of extraction-resistant mRNA in stationary phase *Saccharomyces cerevisiae *produces a massive increase in transcript abundance in response to stress

**DOI:** 10.1186/gb-2006-7-2-r9

**Published:** 2006-02-08

**Authors:** Anthony D Aragon, Gabriel A Quiñones, Edward V Thomas, Sushmita Roy, Margaret Werner-Washburne

**Affiliations:** 1 Department of Biology, University of New Mexico, Albuquerque, NM 87131, USA; 2 Cancer Biology Program, Stanford University, Stanford, CA 94305, USA; 3 Sandia National Laboratories, Albuquerque, NM 87185, USA; 4 Department of Computer Science, University of New Mexico, Albuquerque, NM 87131, USA

## Abstract

**Background:**

As carbon sources are exhausted, *Saccharomyces cerevisiae *cells exhibit reduced metabolic activity and cultures enter the stationary phase. We asked whether cells in stationary phase cultures respond to additional stress at the level of transcript abundance.

**Results:**

Microarrays were used to quantify changes in transcript abundance in cells from stationary phase cultures in response to stress. More than 800 mRNAs increased in abundance by one minute after oxidative stress. A significant number of these mRNAs encode proteins involved in stress responses. We tested whether mRNA increases were due to new transcription, rapid poly-adenylation of message (which would not be detected by microarrays), or potential release of mature mRNA present in the cell but resistant to extraction during RNA isolation. Examination of the response to oxidative stress in an RNA polymerase II mutant, *rpb1-1*, suggested that new transcription was not required. Quantitative RT-PCR analysis of a subset of these transcripts further suggested that the transcripts present in isolated total RNA from stationary phase cultures were polyadenylated. In contrast, over 2,000 transcripts increased after protease treatment of cell-free lysates from stationary phase but not exponentially growing cultures. Different subsets of transcripts were released by oxidative stress and temperature upshift, suggesting that mRNA release is stress-specific.

**Conclusions:**

Cells in stationary phase cultures contain a large number of extraction-resistant mRNAs in a protease-labile, rapidly releasable form. The transcript release appears to be stress-specific. We hypothesize that these transcripts are associated with P-bodies.

## Abstract 2

A rapid transcript increase due to the release of extraction-resistant mRNAs from yeast cells in response to stress is described.

## Background

Quiescence is the most common state of cells on earth [1] and mechanisms for entry into, survival during, and exit from this state are thus likely to be highly conserved. In many cases the signal for quiescence has been identified for both eukaryotes and prokaryotes. For example, in microbes, quiescence is induced in response to various environmental signals. For *Saccharomyces cerevisiae*, the primary signal appears to be carbon starvation [1,2], although other nutrient limitations have been shown to induce a somewhat similar cellular arrest [3]. In more complex eukaryotes, the quiescent state is regulated by hormones and growth regulators and is important both in health, for example, for wound healing and the longevity of cells such as neurons [4] and oocytes [5], and in disease, such as cancer [6] and tuberculosis [7]. Thus, for all organisms, including prokaryotes, the ability to enter, survive in, and exit this state quickly and efficiently provides a selective advantage over evolutionary time [8] and is highly regulated [1].

*S. cerevisiae *cells entering the quiescent state undergo physiological and morphological changes that allow them to survive for long periods of time without added nutrients and passively resist environmental stresses [1,2]. Cells in stationary phase cultures accumulate glycogen and trehalose, develop a thickened cell wall, and become resistant to stresses such as increased temperature and oxidative stress [1,2]. Resistance to temperature stresses can be at least partially explained by the induction of *HSP104 *[9] and to oxidative stress by the accumulation of catalase [10], superoxide dismutase [11] and glutathione [12] soon after the diauxic shift. However, it was not known whether cells in stationary phase cultures could alter transcript abundance in response to additional stress.

Oxidative stress is one of the major stresses encountered by quiescent cells and many genes required for survival in stationary phase encode proteins, such as glutathione transferase and catalase, required for protection from oxidative stress [13]. In the absence of protection from oxidative stress, every type of macromolecule in the cell can be damaged [13]. Paradoxically, mitochondrial activity, which produces free radicals, is essential for survival in stationary phase [14]. Thus, the stress resistance that develops in cells as cultures enter stationary phase must be enough to protect the cell from typical levels of oxidative stress. However, the sensitivity of cells to oxidative stress, the requirement for mitochondrial function, and the reduced rates of transcription [15] and translation [16] in stationary phase that would make a rapid response difficult, might put cells in stationary phase cultures in a precarious position if they were to experience additional stress.

In this study, menadione (2-methyl-1,4-naphthoquinone) was used to generate oxidative stress. Menadione causes oxidative stress through two mechanisms. First, it increases oxidation of NADH and NADPH, resulting in the production of reactive oxygen species (ROS) through redox-cycling [17], which can lead to damage of DNA and other macromolecules [13]. Second, menadione conjugates with the free radical-scavenger glutathione, effectively reducing its concentration [18]. Both maintenance of redox potential and glutathione are known to be essential for survival in stationary phase [14].

In this study we wanted to determine whether cells in stationary phase cultures had an active response to oxidative and temperature stress at the level of changing transcript abundance. Microarray analysis of mRNA isolated from menadione-stressed cells revealed an increase in transcript abundance within one minute of exposure. This response did not require new transcription or poly-adenylation of transcripts. Instead, the full-length mRNAs involved in this response were present in the cell in extraction-resistant, protease-labile complexes. Differences between transcripts released in response to oxidative and temperature stress and after protease treatment further suggested that subsets of transcripts are released in a stress-specific manner.

## Results

### Stationary phase mRNAs exhibit four patterns of response to oxidative stress

To determine whether cells in stationary phase cultures could respond to stress by changes in transcript abundance, cells were harvested at 30 minute time intervals after the addition of 50 μM menadione (final concentration). For time course experiments, total RNA was isolated using the modified Gentra method (see Materials and methods). A carbon source was not added to ensure that metabolic activity would be low and constant and that any changes in transcript abundance would be due to stress and not to re-feeding.

**Figure 1:**
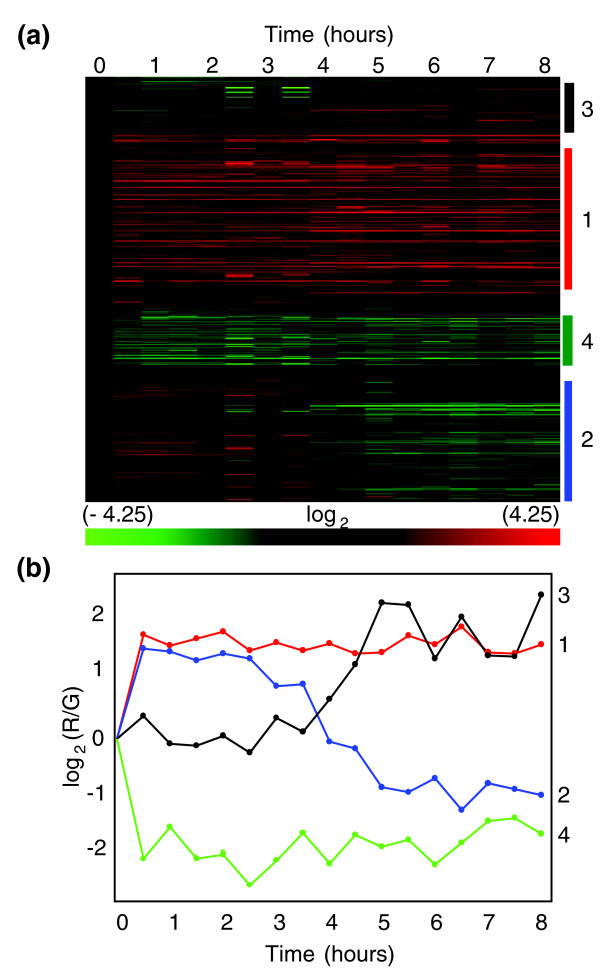
Time course at 30 minute intervals in cells from stationary phase cultures exposed to 50 μM menadione. **(a) **Heat map of results from unsupervised, hierarchical clustering (Pearson's centered, average-linkage) of approximately 2,800 transcripts. Microarrays were of samples taken at 30 minute intervals over 8 hours. The color scale at the bottom indicates the log_2 _values of changes in mRNA abundance. **(b) **Median values for the four major temporal patterns of gene expression identified on the right side of the heat map in (a). RNA was isolated using the modified Gentra method described in Materials and methods.

Four patterns of change in mRNA abundance were observed over the eight hour time course (Figure 1; Additional data file 1). Similar patterns were identified by hierarchical clustering (Figure 1), K-means, and SOM(self-organizing maps) (not shown). By 30 minutes, two groups of transcripts (groups 1 and 2), comprising 1,090 mRNAs, showed 2- to 3-fold increases in abundance (Figure 1b, blue and red lines). Another group of transcripts (group 3) remained unchanged for about three hours and then increased three-fold (Figure 1b, black line). The fourth group of transcripts decreased in abundance (Figure 1b, green line). We concluded from these results that cells in stationary phase cultures could respond to additional environmental stress at the level of transcript abundance and that the response was rapid and relatively complex.

Groups 1 and 2, which increased by the first time point, differed in their patterns of expression over subsequent time points (Figure 1b, blue and red lines). Group 1 (616 transcripts) increased as much as three-fold by the first 30 minute time point and was relatively constant thereafter (Figure 1b, red line). A relatively large percentage (12%, *p *< 10^-4^) of the mRNAs in this group encode proteins with functions related to stress responses, including the bZip transcription factor, *YAP1 *[19] and other proteins associated with oxidative-stress resistance, such as the superoxide dismutase genes *SOD1 *and *SOD2 *[20], thioredoxins *TRX2*, *TSA1*, *PRX1*, *TRR1 *and *TRR2 *[21], and cytochrome-c peroxidase *CCP1 *[22] (Additional data file 1). Transcripts encoded by DNA repair/damage response genes, including *NTG1 *[23], *DNA2 *[24], *DIN7 *[25], were also in this group. Group 2 (474 transcripts) increased by the first time point and began to decrease within two to four hours (Figure 1b, blue line). A large and significant percentage of these transcripts (25%, *p <*10^-10^) encode proteins required for ribosomal biogenesis and processing.

Group 3, comprising 475 transcripts, remained relatively unchanged for the first 4 hours and then gradually increased about 3-fold above T_0 _levels (Figure 1b, black line). A significant number of mRNAs in this group (10%, *p <*10^-4^) encode proteins associated with the proteosome, including ubiquitin (Ubi4p), polyubiquitin [26], Doa1p, which is involved with ubiquitin-mediated protein degradation [27], and the ubiquitin-conjugating enzymes Cdc34p [28] and Rad6p [29] (Additional data file 1). Also in this group are mRNAs that encode components of the proteasome core complex, including *PUP2*, *PUP3*, *SCL1*, and *PRE1-10 *[30].

Group 4, comprising 170 transcripts, decreased approximately 4-fold in abundance within the first 30 minutes and remained constant thereafter (Figure 1b, green line). A significant number of mRNAs in this group (*p <*10^-2^) encode proteins associated with DNA and phospholipid binding (Additional data file 1). We concluded from these results that there are coordinated changes in the abundance of transcripts encoding proteins with a variety of functions, including ribosome processing and biogenesis and response to stress, especially response to oxidative stress.

### Oxidative stress induced a rapid increase in a large number of mRNAs

**Figure 2:**
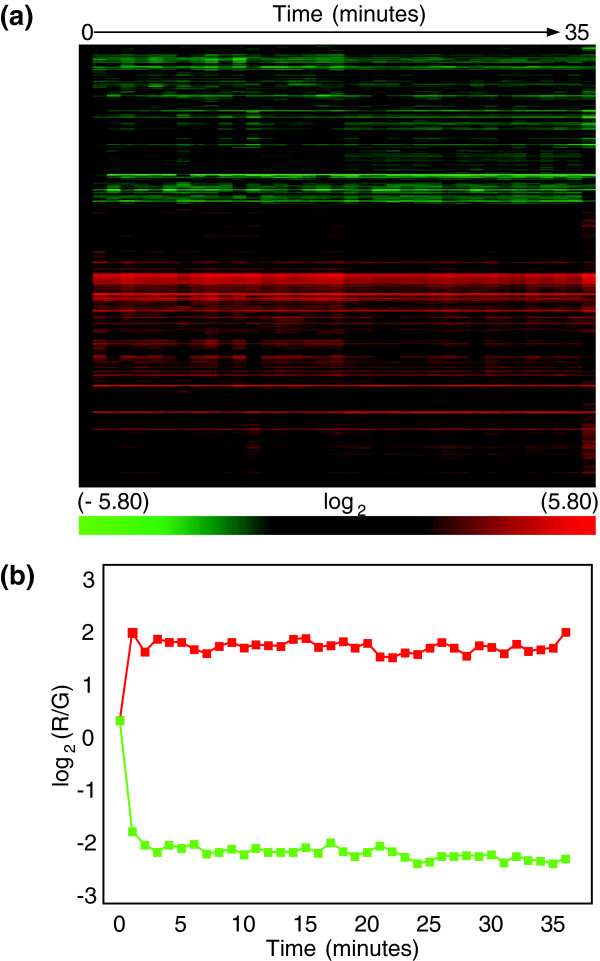
Time course at 1 minute intervals in cells from stationary phase cultures exposed to 50 μM menadione. **(a) **Heat map of results from unsupervised hierarchical clustering (Pearson's centered, average-linkage) of approximately 2,000 transcripts from samples harvested at 1 minute intervals for 35 minutes with an additional sample taken at one hour. **(b) **Median values for the two major temporal patterns of changes in mRNA abundance plotted from the median values of mRNAs clustered in (a). RNA was isolated using the modified Gentra method described in Materials and methods.

To determine the rate of the initial response to oxidative stress, shorter time intervals were needed. To sample more frequently while maintaining culture sterility, we designed and built a pneumatic device that can sample cells in culture at intervals as short as ten seconds [31]. Using this device, cells were harvested at 1 minute time points for the first 35 minutes with an added time point at 1 hour. Surprisingly, a large group of mRNAs increased significantly by the first time point (1 minute) and a much smaller group of mRNAs decreased (Figure 2a; Additional data file 2). After the first time point, transcript abundance remained constant. Analysis of the first time points for both the 30 minute and 1 minute interval time courses revealed 508 transcripts that increased by 2-fold or more in both time courses. We concluded that the same response was detected in both experiments. Because the rate of transcription is known to be very low in cells in stationary phase cultures in the absence of an added carbon source [15] and this response has been shown not to be an artifact of automated sampling [31], the source of these transcripts was puzzling.

We hypothesized the rapid increase in transcripts in response to oxidative stress was due to one or more of three potential mechanisms. First, the apparent increase in transcripts could be due to new transcription. This seemed unlikely because the cells for these experiments were not given a carbon source during the oxidative stress and would be expected to have very low metabolic activity. Second, the transcripts might 'appear' to increase if they lacked a poly(A)^+ ^tail because oligo-dT is used to prime cDNA synthesis for microarrays and, thus, non-polyadenylated transcripts in solution would not be detected. Rapid polyadenylation is known to occur in other systems, including during *Xenopus *oocyte development as well as during the dorsal ventral patterning of the *Drosophila *embryo [32]. A third possibility was that transcripts might not be detected if they were not present in total RNA isolates because they were resistant to extraction, for example, by being in a complex with some structural component in the cell, but were solubilized or released after a stress. This would result in an apparent increase in abundance. If mature RNAs were sequestered in a protein complex, such as stress granules [33] or P-bodies [34], these RNAs would not typically be present in total RNA preparations because one of the first steps in all RNA extraction protocols is selective precipitation of proteins.

### Increased mRNA abundance was not due to *de novo *transcription

**Figure 3:**
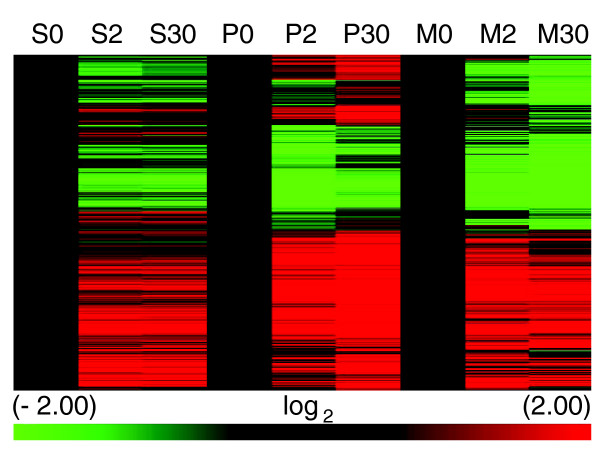
Time course of gene expression in wild type (S), parental (P), and *rpb1-1 *mutant (M) stationary phase cultures exposed to 50 μM menadione for 0, 2, and 30 minutes. Heat map of results from unsupervised hierarchical clustering (Pearson's centered, average-linkage). Approximately 1,000 transcripts were included in this analysis. Samples were taken at T_0_, T_2_, and T_30 _minutes after exposure to 50 μM menadione. The color scale at the bottom indicates the log_2 _values for changes in mRNA abundance.

To determine whether new transcription was responsible for transcript increases, stationary phase cultures of a temperature-sensitive RNA polymerase II mutant (*rpb1-1*) [35], an *RPB1 *parental, and the wild-type S288c strain were incubated for three hours under non-permissive conditions for the *rpb1-1 *mutant (36°C) prior to oxidative stress. By two minutes after menadione exposure, transcript abundance increased dramatically in all three strains (Figure 3). Although there were some differences between these strains, 267 transcripts were identified that increased in all three (Additional data file 3). We concluded from these results that new transcription was an unlikely source for the rapid increase in mRNA after exposure to menadione.

### Increased mRNA abundance was not the result of rapid polyadenylation

**Figure 4:**
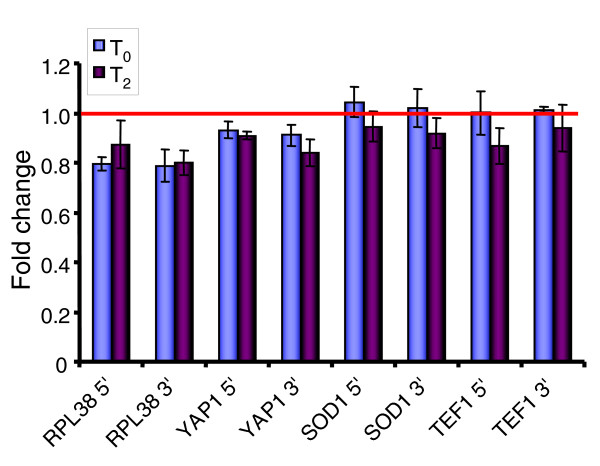
Quantitative RT-PCR analysis to detect presence of non-adenylated transcripts in T_0 _samples and samples 2 minutes after oxidative stress (T_2_). cDNA was synthesized using oligo-dT (to identify polyadenylated transcripts) or random hexamer primers. To determine if 5' or 3' ends of transcripts were more abundant, primer pairs were made to amplify 3' or 5' ends of each of four transcripts. Fold change represents the difference in abundance of 5' or 3' ends of transcripts in cDNAs synthesized using random hexamers versus oligo-dT primers. Measurements were obtained by quantitative RT-PCR and error bars represent the standard deviation of three measurements. The red horizontal bar at Fold Change = 1 indicates no difference in transcript abundance between oligo-dT-primed cDNA and random hexamer-primed cDNA. If non-adenylated transcripts were present, Fold Change > 1 would be expected.

To test the second hypothesis that transcripts were present in isolated total RNA but lacked poly(A)^+ ^tails, we carried out quantitative RT-PCR analysis on cDNA samples synthesized using oligo-dT or random hexamer primers. Oligo-dT would not prime cDNA synthesis from non-adenylated transcripts. To determine whether partial transcripts were present, primer pairs that would amplify small fragments from either 5' or 3' ends of four transcripts (*RPL38*, *TEF1*, *SOD1*, and *YAP1*) that increase significantly after oxidative stress were used. Fold changes between random hexamer-primed and oligo-dT-primed cDNAs were less than one (indicating that more mRNAs were reverse transcribed using oligo-dT primers). In addition, fold changes were not significantly different at T_0 _or T_2 _nor were there major differences in fold change between the 5' or 3' end of any of the four transcripts (Figure 4). We concluded from this that all the transcripts seen at T_0 _and T_2 _were polyadenylated; thus, partial transcripts did not make up a significant percentage of all transcripts. Although a small number of transcripts were evaluated, these results provide evidence that the increase in transcript abundance detected by microarray analysis was not due to the presence of mRNA lacking a poly(A)^+ ^tail but from real differences in mRNA abundance in the isolates.

### Protease treatment of cell free lysates resulted in increased mRNA abundance in cells from stationary phase but not exponential cultures

To test the third hypothesis that the mRNA was present in the cell but resistant to extraction during RNA isolation, cell-free lysates obtained after breaking open cells and precipitating cell debris were tested. Lysates from T_0 _and T_2 _samples (at T = 0 and 2 minutes after menadione exposure) were incubated in buffer or with one of three different proteases, trypsin, proteinase K, or Qiagen protease, for 1 hour at 4°C prior to protein precipitation. Protease digestion was monitored by SDS-PAGE (Additional data file 4). Because the initial cell-free lysate in our RNA isolation protocol is aqueous, it was possible to use protease treatment to determine whether there was protease-labile RNA present. In RNA isolation protocols in which the initial lysis is done with phenol chloroform, protease treatment is not possible.

**Figure 5:**
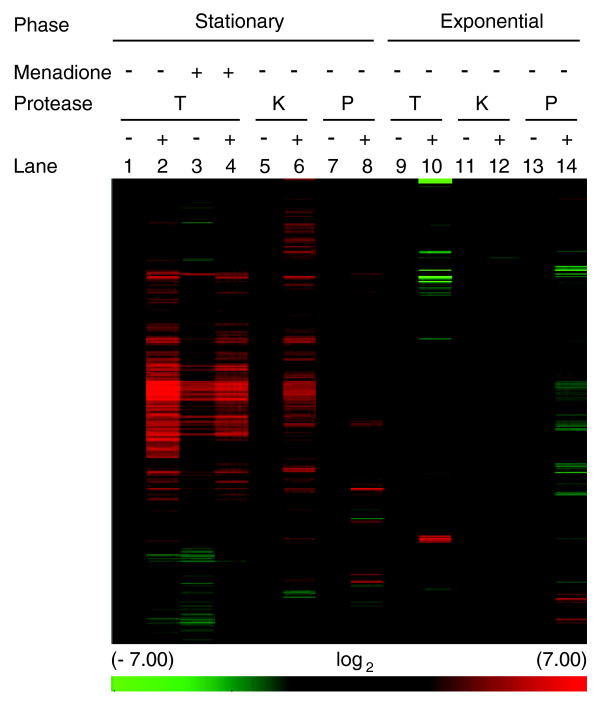
mRNA abundance in samples treated with or without protease. Unsupervised hierarchical clustering (Pearson's centered, average-linkage) of approximately 3,800 transcripts. Samples were incubated with buffer alone (-) or protease (+): trypsin (T), proteinase K (K), Qiagen protease (P). Results were normalized to untreated samples (lanes 1, 5, 7, 9, 11, or 13). Lanes 1 to 8: samples from stationary phase cultures. Lanes 3 and 4: stationary phase samples 2 minutes after treatment with menadione (+). Lanes 9 to 14: exponential samples treated with or without protease. The color scale at the bottom represents the log_2 _values for changes in mRNA abundance.

Approximately 2,100 transcripts from total RNA from T0 samples exhibited 2- to 128-fold increases after trypsin treatment (Figure 5, lanes 1 and 2: Additional data file 5). Many of these same transcripts increased after two minutes of exposure to menadione (without trypsin treatment) (Figure 5, lane 3). However, the increases in transcript abundance after exposure to menadione were less than after trypsin treatment. When T_2 _samples were treated with trypsin, transcripts increased to the level seen in trypsin-treated T_0 _samples (Figure 5, lanes 3 and 4). Digestion of T_0 _samples with proteinase K resulted in similar increases of a larger number of transcripts (Figure 5, lanes 5 and 6), while treatment with Qiagen protease resulted in increases in fewer transcripts (Figure 5, lanes 7 and 8). We suspect that the lower efficiency of Qiagen protease may be a function of substrate specificity. We concluded from this result that RNAs were present in T_0 _cells and by two minutes after menadione exposure most transcripts were in a soluble form. Because these transcripts are released by protease treatment it is likely that they are in complex with protein that is precipitated during most RNA isolation protocols. Finally, because the transcripts released by protease digestion of lysates were detected using oligo-dT primers for cDNA synthesis, we concluded that these transcripts were polyadenylated.

Because most studies of yeast are carried out using exponentially growing cultures, it was of interest to determine whether protease-labile mRNA was also present in dividing cells. Lysates incubated with trypsin, proteinase K, Qiagen protease or buffer only prior to RNA isolation revealed that few, if any, transcripts were in a protease-labile complex in these cells (Figure 5, lanes 9 to 14; Additional data file 5). Of the 16 transcripts that were common to trypsin- and proteinase K-treated lysates, six, *YRB2*, *STV1*, *VPS28*, *DSS4*, *SRO7*, and *SGE1*, encode proteins involved in intracellular transport and establishment of cellular localization. The small group of transcripts that increased after treatment with all three proteases (five genes), suggests that only a few genes are protease labile in these cells. The differences in transcripts that increase after treatment with the three proteases suggests that, if protease-labile, extraction-resistant mRNAs are present in dividing cells, the complexes may be more heterogeneous. Many transcripts show small decreases in abundance with protease treatment that could result from a relatively small change in the mRNA to total RNA ratio in these samples after protease treatment. We concluded from these results that extraction-resistant mRNA, which provides a major pool of mature mRNA in cells from stationary phase cultures, plays a small role, if any, in mRNA pools in non-stressed cells from exponentially growing cultures.

### Hot phenol extraction in the absence of protease treatment does not solubilize the majority of protease-labile mRNA in cells from stationary phase cultures

**Figure 6:**
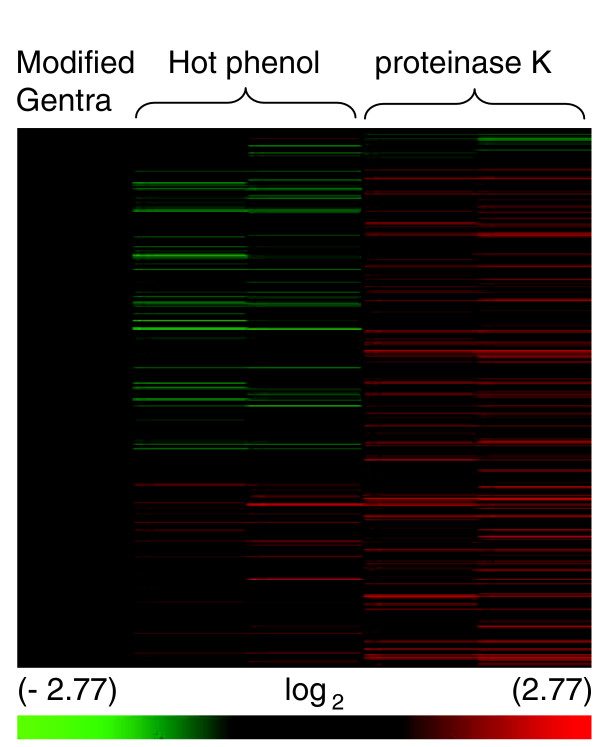
mRNA abundance in samples isolated using two different RNA isolation methods or treated with proteinase K. Unsupervised hierarchical clustering (Person's centered, average-linkage) of approximately 4,000 transcripts. RNA was isolated from unstressed cells from stationary phase cultures using the modified Gentra isolation method, hot phenol, or treated with proteinase K. Results were normalized to samples isolated using our RNA isolation method. Biological replicates for each RNA isolation method are shown. The color scale at the bottom represents the log_2 _values for changes in mRNA abundance.

Although our RNA isolation protocol, which includes an initial aqueous lysis step, allowed us to treat the lysates with proteases prior to protein precipitation, it was possible that a potentially more disruptive RNA isolation technique might solubilize these transcripts in the absence of protease treatment. To test this hypothesis we isolated total RNA from unstressed cells from stationary phase cultures using hot phenol and compared these results with RNAs isolated after trypsin treatment. Transcript abundance was quantified using both microarray analysis (Figure 6) and quantitative RT-PCR (Additional data file 6). When compared with our RNA isolation protocol, replicate analysis mRNA extracted with hot phenol revealed that a small percentage of transcripts were solubilized more effectively with hot phenol while a larger percentage of mRNA was isolated more efficiently in our protocol. We concluded, after comparing mRNAs isolated by either protocol with transcripts isolated after proteinase K treatment, that neither protocol was as effective as the isolation including protease treatment. Quantitative RT-PCR corroborated these findings for a small number of transcripts (Additional data file 6). Although neither standard RNA isolation protocol was as effective as proteinase treatment, the differences between the two extraction protocols suggest that transcripts may bind to intracellular components with very different affinities and be extracted differentially by the two procedures.

### mRNA released in response to oxidative stress was a subset of protease-labile mRNA

**Figure 7:**
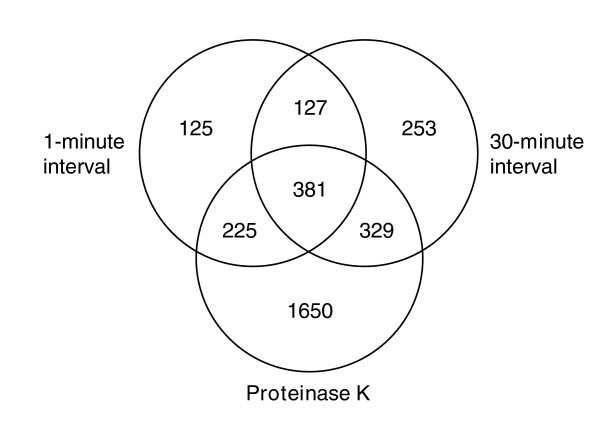
Venn diagram of transcripts that increased after oxidative stress or proteinase K treatment of T_0 _cell lysates. Transcripts were evaluated that had a ≥2-fold increase in abundance by 1 and 30 minutes after oxidative stress or after proteinase K treatment. Transcripts were also required to have good spots in 80% of the time points.

To determine the overlap between transcripts released after oxidative stress and proteinase K treatment, we compared transcripts from 30 minute and 1 minute oxidative stress time courses and after proteinase treatment of T_0 _samples (Figure 7). Although there were transcripts exclusive to each treatment, 65% of all transcripts that increased in response to oxidative stress also increased after proteinase K treatment. For both the 30 and 1 minute time courses, a significant number of transcripts that increased in response to oxidative stress also increased after proteinase K treatment (*p <*1 × 10^-15 ^for both). In contrast, only 45% of the transcripts that increased after proteinase K treatment of unstressed cells from stationary phase cultures also increased in response to oxidative stress. This is consistent with the earlier observation that many transcripts are present in a protease-labile form in cells two minutes after oxidative stress (Figure 5, lanes 3 and 4) and suggests that sequestered transcripts are present in these cells 30 minutes after oxidative stress.

### mRNA release was stress specific

**Figure 8:**
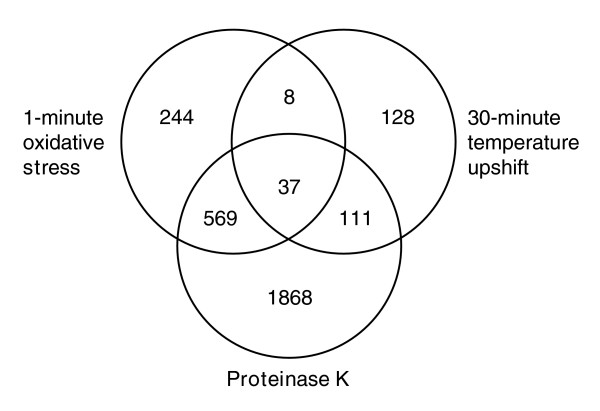
Venn diagram of transcripts that increased by 1 minute after oxidative stress, 30 minutes after temperature upshift, or after proteinase K treatment of T_0 _cell lysates. Transcripts used for this analysis were filtered as described in Figure 7.

Finally, to examine whether mRNA released from protein complexes is stress specific, we determined the overlap between transcripts that increased at least two-fold after oxidative stress (1 minute), temperature upshift (30°C to 39°C for 30 minutes), and proteinase K treatment (Figure 8) of stationary phase samples. As seen above (Figure 7), a significant percentage of transcripts that increased after oxidative stress also increased after proteinase K treatment. Likewise, a significant percentage (52%, *p* = 9.58 × 10^-5^) of transcripts that increased after a temperature upshift (Additional data file 7) also increased after proteinase K treatment. Interestingly, only 45 transcripts were found to increase in response to both oxidative stress and a temperature upshift. This represented 16% and 5% of transcripts increased in response to temperature upshift and oxidative stress, respectively. The *p *value of 0.28 for this overlap indicated that transcripts released in response to oxidative stress and by temperature upshift showed no significant similarity. Consistent with earlier results, only 28% of proteinase K-labile transcripts increased in response to either stress; that is, 72% of the transcripts that increased in response to proteinase K treatment were not increased after either stress. From these results we concluded that: there is a large pool of protease-labile mRNA that are not released in response to either of the stresses tested here and that may be released in response to other environmental signals, such as re-feeding [14]; and protease labile mRNAs appear to be released from protein-mRNA complexes in a stress specific manner.

## Discussion

We found that yeast cells in stationary phase cultures exhibited a rapid, non-transcriptional response to oxidative stress resulting in the apparent increase in abundance of hundreds of transcripts. These mRNAs were present as intact messages that appear to be bound to protein in cells in stationary phase cultures. The extraction-resistant transcripts encode proteins that are known to be induced after oxidative stress, DNA repair, and ribosome processing and assembly. Although there is little translation occurring at this time [16], it seems likely that this response could lead to more rapid translation of stress-response proteins if a carbon source becomes available.

Rapid changes in transcript abundance in cells from stationary phase cultures have also been reported in response to other environmental signals. For example, by the first 5 or 10 minute time point after re-feeding cells in stationary phase cultures, there were significant increases in over 1,000 transcripts [14,36]. The major overlaps in RNAs that increased during both exit from stationary phase and in response to oxidative stress encoded ribosome processing proteins and ribosomal proteins. This response has not yet been shown to result from release of extraction-resistant mRNA and it was recently reported that inactive RNA polymerase II is positioned on many genes that are induced early during exit from stationary phase [37]. We believe that the rapid response in cells from stationary phase cultures after re-feeding is likely to be a combination of very fast transcript release and subsequent transcription. We hypothesize that specific transcript release in cells in stationary phase cultures is a mechanism to allow cells that have very low metabolic rates to respond as quickly as possible to environmental conditions, in preparation for the activation of transcription and translation.

Interestingly, there is a 40% overlap between transcripts that increase by 1 minute in this study and transcripts that increase by the first time point (10 minutes) after oxidative stress in exponential cells [38]. Because we found little evidence for extraction resistant mRNA in exponential cells, the rapid increase in mRNA abundance in the study of Gasch *et al. *[38] is likely the result of transcription. We hypothesize, based on the overlap between these data sets and the observation that RNA polymerase II is positioned on the promoters of many of these genes in stationary phase [37], that the cell is programmed to increase the abundance of this group of transcripts in response to stress under any condition. When cells are growing, increased transcription would lead to induction of these transcripts, whereas in stressed cells, sequestration and transcript release would allow increases in 'apparent' transcript abundance.

Previously, increases in transcript abundance in response to stress have always been assumed to be the result of transcription and decreases the result of decreased transcription and/or increased turnover. For example, when transcripts decrease after the diauxic shift or when the Tor pathway is inhibited, it was assumed to result from mRNA degradation [39,40]. mRNA sequestration has not generally been considered to be a major factor in the dynamics of mRNA abundance. Approximately half of the transcripts that increase after proteinase K treatment of T_0 _samples also decrease after the diauxic shift as cultures enter stationary phase [37,38]. Thus, our results provide another hypothesis for these observations, that inactivation of TOR1 or cAMP-dependent protein kinase (PKA) or both, which is required at the diauxic shift during the post-diauxic phase [1] stimulates the accumulation of mRNA in RNA-protein complexes in stationary phase cultures.

Two known protein-mRNA complexes could be involved in mRNA sequestration in cells from stationary phase cultures: stress granules and P-bodies. Stress granules, not previously identified in *S. cerevisiae*, are protein-RNA complexes that accumulate in response to various stresses, including oxidative stress in mammalian and plant cells [33]. These granules, which have been studied primarily microscopically, are cytoplasmic, accumulate pre-initiation complexes that contain as much as 50% of the total poly(A)^+ ^RNA in a cell, and decrease in abundance within 60 to 90 minutes when translation rates increase and conditions become favorable for growth [41,42].

P-bodies have been identified in *S. cerevisiae *and accumulate in yeast cells in stationary phase cultures [43]. Although P-bodies are generally considered to be sites of mRNA decapping and deadenylation [44], they have been hypothesized to be sites of mRNA storage [34]. P-bodies are similar to mammalian stress granules in that there is a direct correlation between their appearance and abundance and the rate of translation. P-bodies have recently been shown to increase significantly in size as cultures enter stationary phase [43] and contain mRNA that can be released from protein complexes and re-enter translation [45]. Thus, it is likely that P-bodies are the site of mRNA sequestration in cells in stationary phase cultures. Although these two complexes have been studied for many years, this is the first genomic evidence for the extent and specificity of mRNA sequestration in P-bodies in yeast and the rate at which transcripts can be released from these complexes.

In conclusion, the ability of yeast cells in stationary phase cultures to respond to stress in the absence of added nutrients by releasing extraction resistant mRNAs provides important insight into the physiology of quiescent cells and the dynamics and regulation of stress responses. It also leads to further questions about the specificity, regulation, and development of this response. Finally, it underscores the potential significance of investigating cellular processes and responses during other, less-studied stages of the life cycle.

## Materials and methods

### Website

Supplemental information and more detailed protocols are available at [46].

### Growth conditions

*MATα *S288c (*his3 leu2-3*, *112 lys2 trp1 ura3-52*) cells were grown in YPD+A (1% yeast extract, 2% peptone, 2% D-glucose, and 0.04 mg/ml adenine) with aeration at 30°C for 7 days (to OD_600 _20-24). Rapidly dividing (exponential) cells were collected after overnight growth (OD_600 _1-2) under the same conditions.

### Cell harvesting

Cells from stationary phase and exponentially growing cultures were collected to serve as a common reference for all experiments. Several cell samples were taken prior to exposure to menadione to serve as a T_0 _reference. For oxidative stress menadione was added from a 500 μM stock to a final concentration of 50 μM. For the 30 minute time interval time course, cells were collected by pipette every 30 minutes for 8 hours. Cell viability was constant for at least 8 hours after exposure to this concentration of menadione (data not shown). For the second time course, an automated-sampling device [31] was used to collect cells at 1 minute intervals for 35 minutes, with a final time point at 1 hour. For both time courses, cells were harvested and analyzed in duplicate by microarrays. For temperature upshift, cultures were grown to stationary phase at 30°C, shifted to 39°C, and cells harvested by pipette every 30 minutes for 8 hours.

### Experimental design

A random block design [47] was used to eliminate artificial sources of periodicity that may be introduced due to specific, constant ordering of time course samples during RNA isolation, cDNA labeling or hybridization, as well as to avoid confounding factors throughout the experiment. In a randomized block design, each time course is treated as a single block and samples within a time course were randomized for RNA isolation and re-randomized for both cDNA labeling and hybridization.

### RNA isolation

For RNA isolation (Additional data file 8), a modified Gentra protocol was used. Briefly, 20 OD_600 _of cells from exponential phase or 30 OD_600 _of cells from stationary phase cultures were lysed at 4°C in 300 μl of Cell Lysis Solution (Gentra, Minneapolis, MN, USA) using 0.5 mm glass beads (Sigma, St Louis, MO, USA) and a mechanical bead beater (Biospec Products, Bartlesville, OK, USA) at 4,800 rpm. Lysis was carried out in 6, 30-second bursts alternating with 30 seconds on ice. Samples were spun at 13,000 × *g *for 3 minutes at 4°C. After centrifugation, 100 μl of Protein-DNA Precipitation Solution (Gentra) was added to the supernatant and samples were incubated on ice for 5 minutes. Protein-DNA precipitate was pelleted at 13,000 × *g *for 3 minutes at 4°C. RNA was precipitated using 300 μl 100% isopropanol and pelleted by centrifugation. After centrifugation, RNA was resuspended in DEPC (diethylpyrocarbonate)-treated H_2_O and a phenol-chloroform (5:1) 'back extraction' was performed (Ambion, Austin, TX, USA). RNA was precipitated overnight in 0.1 volume of 0.5 M NH_4_OAc and 2.5 times the volume of 100% ethanol and subsequently purified using a Qiagen RNeasy kit (Qiagen, Alameda, CA, USA). RNA quality was evaluated using a Bioanalyzer 2100 (Agilent, Palo Alto, CA, USA).

For hot phenol extractions, 30 OD_600 _of cells from stationary phase cultures were resuspended in 1 ml of sodium acetate buffer (50 mM sodium acetate, 10 mM EDTA, 0.1% SDS) and an equal amount of saturated phenol (Fisher Scientific, Pittsburgh, PA, USA) at 65°C. Samples were incubated at 65°C for 10 minutes, with vortexing every 1 minute for 10 seconds. Samples were spun for 10 minutes. The aqueous phase was then transferred to 1 ml of saturated phenol, vortexed for 1 minute and spun for 10 minutes. After the previous step was repeated the aqueous phase was transferred to 1 ml of chloroform (Sigma), vortexed for 45 seconds, and spun for 5 minutes. RNA was precipitated with 0.1 volume of 3 M Na Ac pH 5.2 and 2 times the volume of 100% ethanol at -20°C for 1 hour. RNA was pelleted by centrifugation, washed with 70% ethanol, and RNA was resuspended in DEPC-treated H_2_O. RNA quality was evaluated using a Bioanalyzer 2100 (Agilent).

### Array printing and slide treatment

UltraGAPS slides (Corning, Corning, NY, USA) were printed using an OmniGrid 100 Arrayer (GeneMachines, San Carlos, CA, USA) with SMP4 printing pins (TeleChem, Sunnyvale, CA, USA). The yeast genomic oligonucleotide set (containing 70-mers corresponding to 6,307 open reading frames; Qiagen) was resuspended in 3× SSC to a final oligonucleotide concentration of 40 μM, and used to print the slides.

During printing, the relative humidity was maintained between 50% and 52% and the ambient temperature was maintained between 21°C and 23°C. After printing, slides were UV cross-linked at 90 mJ in a UV Stratalinker 1800 (Stratagene, La Jolla, CA, USA) and baked at 80°C overnight. For validation and quality control, slides were scanned after pre-hybridization treatment (see below) to screen for spot-localized contamination [48]; SYBR green II staining (Invitrogen, Carlsbad, CA, USA) was used to test for DNA binding; and reproducibility experiments to test slide-to-slide reproducibility were carried out prior to and in each experiment. Typically, slide-to-slide standard deviation was in the range of 0.08 log_2 _units for expression ratios.

### Preparation of labeled cDNA

A modified direct-labeling protocol [49] was used to fluorescently label cDNA with Cy3-dCTP or Cy5-dCTP (Amersham Biosciences, Piscataway, NJ, USA) using the Corning Microarray Technologies (CMT) Yeast Array 9/00 protocol (Corning; Additional data file 9). Total RNA (20 μg) from experimental samples were reverse transcribed to cDNA labeled with Cy5. A common reference sample RNA (20 μg of stationary phase and exponential RNAs combined at a 1:1 ratio) was reverse transcribed to cDNA labeled with Cy3 and pooled after labeling. A pooled sample of the common reference was optimized to maximize the number of genes hybridized and reduce variability throughout the array [50] and was used for all experiments. The use of a common reference allows all experimental information to be analyzed in relationship to the same reference, allowing better normalization [51].

For within-slide normalization, 10 ng of *Arabidopsis thaliana CAB *mRNA (Stratagene) was added to each labeling reaction to be used. Because we use a common reference and all the experimental information is in Cy5-labeled cDNA, even if the CAB mRNA labels slightly differently using Cy3 or Cy5, the normalization is consistent throughout the experiment.

### Pre-hybridization and hybridization

Slides were pre-hybridized in a 250 ml glass Coplin jar for 1 to 2 hours at 42°C in a freshly prepared solution containing 50% formamide, 5× SSC, 0.1% SDS and 0.1 mg/ml bovine serum albumin fraction V (Sigma; Additional data file 10). The slides were rinsed several times with ddH_2_O, dipped in 100% ethanol, and dried under a 30 psi stream of N_2 _gas. Prior to hybridization, 22 × 30 mm Lifterslip coverslips (Erie Scientific, Portsmouth, NH, USA) were cleaned in a solution of 1 M KOH and 50% ethanol, rinsed with ddH_2_O and dried with 30 psi of N_2 _gas.

The hybridization buffer contained 50% formamide (Sigma), 5% dextran sulfate (Sigma), 5× SSC, 0.1% SDS, 0.1 mg/ml bovine serum albumin fraction V (Sigma), and 100 μg/ml salmon sperm DNA (Invitrogen). For all hybridizations, reference samples were labeled and pooled prior to hybridization. Each labeled experimental sample was combined with an aliquot of the labeled reference sample and dried down in a vacuum centrifuge.

The combined reactions for each slide were resuspended in 35 μl hybridization buffer, incubated at 95°C for 5 minutes, centrifuged for 30 seconds, and applied to the center of the coverslip that was subsequently positioned to cover the printed section of the slide. Slides were sealed in CMT hybridization chambers (Corning) and incubated at 42°C for at least 16 hours on a rocking platform. After hybridization, slides were washed as previously described [48] and subsequently dried using a 30 psi stream of N_2 _gas.

### Microarray scanning and data analysis

All scans were performed with 100% laser power and photomultiplier tube (PMT) settings of 630 to 700 for the 635 nm laser and 430 to 500 for the 532 nm laser using Axon 4000B (Axon Instruments, Union City, CA, USA). These settings provided the maximum signal to noise ratio while reducing the number of saturated spots on each array. Grids used to define spot circumference, location, and identity were made and aligned using GenePix Pro 6.0 (Axon Instruments). All microarray data have been submitted to Gene Expression Omnibus series accession number GSE3729.

### Evaluation of correlation between biological and technical replicates

An ANOVA measurement model was used to determine variance between six independent biological and technical replicates of T_0 _samples (Additional data file 11). The analysis showed a standard deviation for log_2 _ratios of 0.08 for both biological and technical replicates, indicating that a change in gene expression greater than 1.6-fold could be viewed as statistically significant with a Type I error (or false positive rate) of ≤ 0.01.

### Quantitative RT-PCR analysis

Primers were designed using Primer Express (ABI, Foster City, CA, USA) and total RNA was reverse transcribed using either oligo-dT or random hexamers as primers. The two types of primers were used to determine whether transcripts lacking a poly(A)+ tail were present in total RNA isolated from unstressed, stationary phase samples. Quantitative RT-PCR reactions contained SYBR Green PCR Master Mix (ABI), forward and reverse primers (0.6 μM each), and a cDNA template (10 ng). Reactions were done in triplicate (technical replicates) for each sample and exogenous *A. thaliana *RNA (Stratagene) was used as a control (Additional data file 12). The cycle threshold (C_T_) value for each reaction was determined using the ABI Prism 7000 SDS software package (ABI). C_T _values were used to calculate the mean fold change of the reactions via the  method, for which 1 indicates no change in abundance [52]. For this experiment, the mean fold change was the difference in abundance in random hexamer-primed cDNA compared with the abundance in oligo-dT-primed cDNA.

### Protease treatments

After bead beating samples were centrifuged at 13,000 × *g *for 3 minutes at 4°C to remove cell debris. Cell-free lysates were divided into 2 tubes and incubated on ice for 1 hour with either 17 mg/ml trypsin in 0.9% (w/v) NaCl (Invitrogen), 14 mg/ml proteinase K in 10 mM Tris pH 7.5 (Qiagen), 11 mg/ml Qiagen protease in 10 mM Tris pH 7.5 (Qiagen), or 10 mM Tris pH 7.5 alone. After incubation, the RNA isolation for both samples was completed as described above. To visually evaluate the extent of protein digestion under these conditions, proteins isolated from protease-treated and control samples were separated by electrophoresis on a 10% TBE polyacrylamide gel (Bio-Rad, Hercules, CA, USA; Additional data file 4). All experiments were done in duplicate, using biological replicates. Microarray analysis showed that RNA isolated from samples incubated in buffer for 1 hour at 4°C were essentially identical to samples processed immediately (R^2 ^= 0.97).

### RNA polymerase II mutant

*MAT*a (*ura3-52 rpb1-1*) RNA polymerase II temperature-sensitive mutant [35], parental *MATα *(*ura3-52*), and wild-type S288c cells were grown to stationary phase (10 days) with aeration in 100 ml of YPD+A at 24°C. Prior to exposure to menadione, all strains were incubated for 3 hours at 36°C (non-permissive conditions for *rpb1-1*) prior to the collection of T_0 _samples. Oxidative stress was induced using menadione as described above and samples from *rpb1-1*, parental and S288c cultures were harvested at 2 and 30 minutes after exposure.

### Statistical analysis of transcript abundance

A normal approximation to Fisher's exact test [53] was used to generate a *p* value for testing the association between membership on two sets of gene lists.

## Additional data files

The following additional data are included with the online version of this article. Additional data file 1 is a gene list from the 30 minute interval time course. Additional data file 2 is a gene list from the 1 minute interval time course. Additional data file 3 is a gene list from the polymerase II mutant data set. Additional data file 4 contains SDS-PAGE images. Additional data file 5 provides gene lists from the protease treatments. Additional data file 6 is a graph of the quantitative RT-PCR experiments. Additional data file 7 is a gene list from the temperature upshift experiment. Additional data file 8 describes in detail the RNA isolation protocol. Additional data file 9 describes in detail the labeling protocol. Additional data file 10 describes in detail the hybridization protocol. Additional data file 11 provides a detailed description of the ANOVA measurement model. Additional data file 12 describes in detail the quantitative RT-PCR protocol.

## Supplementary Material

Additional data file 1 Gene list from 30 minute interval time course. Click here for file

Additional data file 2 Gene list from 1 minute interval time course. Click here for file

Additional data file 3 Gene list from polymerase II mutant data set. Click here for file

Additional data file 4 SDS-PAGE images. Click here for file

Additional data file 5 Gene lists from protease treatments. Click here for file

Additional data file 6 Graph of quantitative RT-PCR. Click here for file

Additional data file 7 Gene list from temperature upshift. Click here for file

Additional data file 8 Detailed description of the RNA isolation protocol. Click here for file

Additional data file 9 Detailed description of the labeling protocol. Click here for file

Additional data file 10 Detailed description of the hybridization protocol. Click here for file

Additional data file 11 Detailed description of the ANOVA measurement model. Click here for file

Additional data file 12 Detailed description of the quantitative RT-PCR protocol. Click here for file

## References

[B1] Gray JV, Petsko GA, Johnston GC, Ringe D, Singer RA, Werner-Washburne M (2004). "Sleeping beauty": Quiescence in *Saccharomyces cerevisiae*.. Microbiol Mol Biol Rev.

[B2] Werner-Washburne M, Braun E, Johnston GC, Singer RA (1993). Stationary phase in the yeast *Saccharomyces cerevisiae*.. Microbiol Rev.

[B3] Giots F, Donaton M, Thevelein J (2003). Inorganic phosphate is sensed by specific phosphate carriers and acts in concert with glucose as a nutrient signal for activation of the protein kinase A pathway in the yeast *Saccharomyces cerevisiae*. Mol Microbiol.

[B4] Wolkow CA (2002). Life span: getting the signal from the nervous system.. Trends Neurosci.

[B5] Bevers MM, Izadyar F (2002). Role of growth hormone and growth hormone receptor in oocyte maturation.. Mol Cell Endocrinol.

[B6] Fu LN, Lee CC (2003). The circadian clock: Pacemaker and tumour suppressor.. Nature Reviews Cancer.

[B7] Parrish NM, Dick JD, Bishai WR (1998). Mechanisms of latency in *Mycobacterium tuberculosis*.. Trends Microbiol.

[B8] Vulic M, Kolter R (2001). Evolutionary cheating in *Escherichia coli *stationary phase cultures.. Genetics.

[B9] Sanchez Y, Taulien J, Borkovich KA, Lindquist S (1992). Hsp104 is required for tolerance to many forms of stress.. EMBO J.

[B10] Bissinger PH, Wieser R, Hamilton B, Ruis H (1989). Control of *Saccharomyces cerevisiae *catalase T gene (*CTT1*) expression by nutrient supply via the RAS-cyclic AMP pathway.. Mol Cell Biol.

[B11] Longo V, Gralla EB, Valentine JS (1996). Superoxide dismutase activity is essential for stationary phase survival in *Saccharomyces cerevisiae*.. J Biol Chem.

[B12] Grant CM, Collinson LP, Roe J-H, Dawes IW (1996). Yeast glutathione reductase is required for protection agains oxidative stress and is a target gene for yAP-1 transcriptional regulation.. Mol Microbiol.

[B13] Jamieson DJ (1998). Oxidative stress responses of the yeast *Saccharomyces cerevisiae*.. Yeast.

[B14] Martinez MJ, Roy S, Archuletta AB, Wentzell PD, Santa Anna-Arriola S, Rodriguez AL, Aragon AD, Quinones GA, Allen C, Werner-Washburne M (2004). Genomic analysis of stationary phase and exit in *Saccharomyces cerevisiae*: Gene expression and identification of novel essential genes.. Mol Biol Cell.

[B15] Paz I, Meunier JR, Choder M (1999). Monitoring dynamics of gene expression in yeast during stationary phase.. Gene.

[B16] Fuge EK, Braun EL, Werner-Washburne M (1994). Protein-synthesis in long-term stationary phase cultures of *Saccharomyces cerevisiae*.. J Bacteriol.

[B17] Gutierrez PL (2000). The metabolism of quinone-containing alkylating agents: Free radical production and measurement.. Front Biosci.

[B18] Zadzinski R, Fortuniak A, Bilinski T, Grey M, Bartosz G (1998). Menadione toxicity in *Saccharomyces cerevisiae* cells: Activation by conjugation with glutathione.. Biochem Mol Biol Int.

[B19] Kuge S, Jones N, Nomoto A (1997). Regulation of yAP-1 nuclear localization in response to oxidative stress.. EMBO J.

[B20] Maris AF, Assumpcao ALK, Bonatto D, Brendel M, Henriques JAP (2001). Diauxic shift-induced stress resistance against hydroperoxides in *Saccharomyces cerevisiae *is not an adaptive stress response and does not depend on functional mitochondria.. Curr Genet.

[B21] Carmel-Harel O, Storz G (2000). Roles of the glutathione- and thioredoxin-dependent reduction systems in the *Escherichia coli *and *Saccharomyces cerevisiae *responses to oxidative stress.. Annu Rev Microbiol.

[B22] Kwon M, Chong S, Han S, Kim K (2003). Oxidative stresses elevate the expression of cytochrome c peroxidase in *Saccharomyces cerevisiae*.. Biochim Biophys Acta.

[B23] Maclean MJ, Aamodt R, Harris N, Alseth I, Seeberg E, Bjoras M, Piper PW (2003). Base excision repair activities required for yeast to attain a full chronological life span.. Aging Cell.

[B24] Lee KH, Kim DW, Bae SH, Kim JA, Ryu GH, Kwon YN, Kim KA, Koo HS, Seo YS (2000). The endonuclease activity of the yeast *DNA2 *enzyme is essential *in vivo*.. Nucleic Acids Res.

[B25] Fikus MU, Mieczkowski PA, Koprowski P, Rytka J, Sledziewska-Gojska E, Ciesla Z (2000). The product of the DNA damage-inducible gene of *Saccharomyces cerevisiae, DIN7*, specifically functions in mitochondria.. Genetics.

[B26] Finley D, Özaynak E, Varshavsky A (1987). The yeast polyubiquitin gene is essential for resistance to high-temperatures, starvation, and other stresses.. Cell.

[B27] Wilson TE (2002). A genomics-based screen for yeast mutants with an altered recombination/end joining repair ratio.. Genetics.

[B28] Goebl MG, Yochem J, Jentsch S, McGrath JP, Varshavsky A, Byers B (1988). The yeast cell cycle gene *CDC34 *encodes a ubiquitin-conjugating enzyme.. Science.

[B29] Bailly V, Lauder S, Prakash S, Prakash L (1997). Yeast DNA repair proteins *RAD6 *and *RAD18 *form a heterodimer that has ubiquitinconjugating, DNA binding, and ATP hydrolytic activities.. J Biol Chem.

[B30] Voges D, Zwickl P, Baumeister W (1999). The 26S proteasome: A molecular machine designed for controlled proteolysis.. Annu Rev Biochem.

[B31] Aragon AD, Quinones GA, Allen C, Thomas J, Roy S, Davidson GS, Wentzell PD, Millier P, Jaetao JA, Rodriguez AL (2005). An automated, pressure-driven sampling device for harvesting from liquid cultures for genomic and biochemical analyses.. J Microbiological Methods.

[B32] Richter JD (1999). Cytoplasmic polyadenylation in development and beyond.. Microbiol Mol Biol Rev.

[B33] Kedersha N, Anderson P (2002). Stress granules: sites of mRNA triage that regulate mRNA stability and translatability.. Biochem Soc Trans.

[B34] Coller J, Parker R (2004). Eukaryotic mRNA decapping.. Annu Rev Biochem.

[B35] Nonet M, Scafe C, Sexton J, Young R (1987). Eucaryotic RNA polymerase conditional mutant that rapidly ceases mRNA synthesis.. Mol Cell Biol.

[B36] Newcomb L, Diderich J, Slattery M, Heideman W (2003). Glucose regulation of *Saccharomyces cerevisiae *cell cycle genes.. Eukaryotic Cell.

[B37] Radonjic M, Andrau JC, Lijnzaad P, Kemmeren P, Kockelkorn TTJP, van Leenen D, van Berkum NL, Holstege FCP (2005). Genome-wide analyses reveal RNA polymerase II located upstream of genes poised for rapid response upon *S. cerevisiae *stationary phase exit.. Mol Cell.

[B38] Gasch AP, Spellman PT, Kao CM, Carmel-Harel O, Eisen MB, Storz G, Botstein D, Brown PO (2000). Genomic expression programs in the response of yeast cells to environmental changes.. Mol Biol Cell.

[B39] Choder M (1991). A general topoisomerase I-dependent transcriptional repression in the stationary phase of yeast.. Genes Dev.

[B40] Albig A, Decker C (2001). The target of rapamycin signaling pathway regulates mRNA turnover in the yeast *Saccharomyces cerevisiae*.. Mol Biol Cell.

[B41] Anderson P, Kedersha N (2002). Stressful initiations.. J Cell Sci.

[B42] Kedersha N, Cho MR, Li W, Yacono PW, Chen S, Gilks N, Golan DE, Anderson P (2000). Dynamic shuttling of TIA-1 accompanies the recruitment of mRNA to mammalian stress granules.. J Cell Biol.

[B43] Teixeira D, Sheth U, Valencia-Sanchez MA, Brengues M, Parker R (2005). Processing bodies require RNA for assembly and contain nontranslating mRNAs.. RNA.

[B44] Sheth U, Parker R (2003). Decapping and decay of messenger RNA occur in cytoplasmic processing bodies.. Science.

[B45] Brengues M, Teixeira D, Parker R (2005). Movement of eukaryotic mRNAs between polysomes and cytoplasmic processing bodies.. Science.

[B46] Extraction-resistant mRNAs from Stationary phase Cultures. http://biology.unm.edu/biology/maggieww/Public_Html/aragon/ROX.htm.

[B47] Addelman S (1969). The generalized randomized block design.. Am Statistician.

[B48] Martinez MJ, Aragon AD, Rodriguez AL, Weber JM, Timlin JA, Sinclair MB, Haaland DM, Werner-Washburne M (2003). Identification and removal of contaminating fluorescence from commercial and in-house printed DNA microarrays.. Nucleic Acids Res.

[B49] Hegde P, Qi R, Abernathy K, Gay C, Dharap S, Gaspard R, Hughes JE, Snesrud E, Lee N, Quackenbush J (2000). A concise guide to cDNA microarray analysis.. Biotechniques.

[B50] Sterrenburg E, Turk R, Boer JM, van Ommen GB, den Dunnen JT (2002). A common reference for cDNA microarray hybridizations.. Nucleic Acids Res.

[B51] Yang YH, Dudoit S, Luu P, Lin DM, Peng V, Ngai J, Speed TP (2002). Normalization for cDNA microarray data: a Robust composite method addressing single and multiple slide systematic variation.. Nucleic Acids Res.

[B52] Livak KJ, Schmittgen TD (2001). Analysis of relative gene expression data using real-time quantitative PCR and the 2(T)(-Delta Delta C) method.. Methods.

[B53] Fisher RA (1935). The logic of inductive inference.. J Roy Stat Soc Series A.

[B54] US Department of Energy Genomics: GTL Program. http://www.doegenomestolife.org.

[B55] Genomes to Life Project. http://www.genomes2life.org.

[B56] Cleveland WS (1979). Robust locally weighted regression and smoothing scatterplots.. J Am Stat Assoc.

