# Supplementary material for: Release of extraction-resistant mRNA in stationary phase Saccharomyces cerevisiae produces a massive increase in transcript abundance in response to stress
Source: Genome Biol. 2006 Feb 8;7(2):R9. doi: 10.1186/gb-2006-7-2-r9 (PMC1431719; doi:10.1186/gb-2006-7-2-r9)

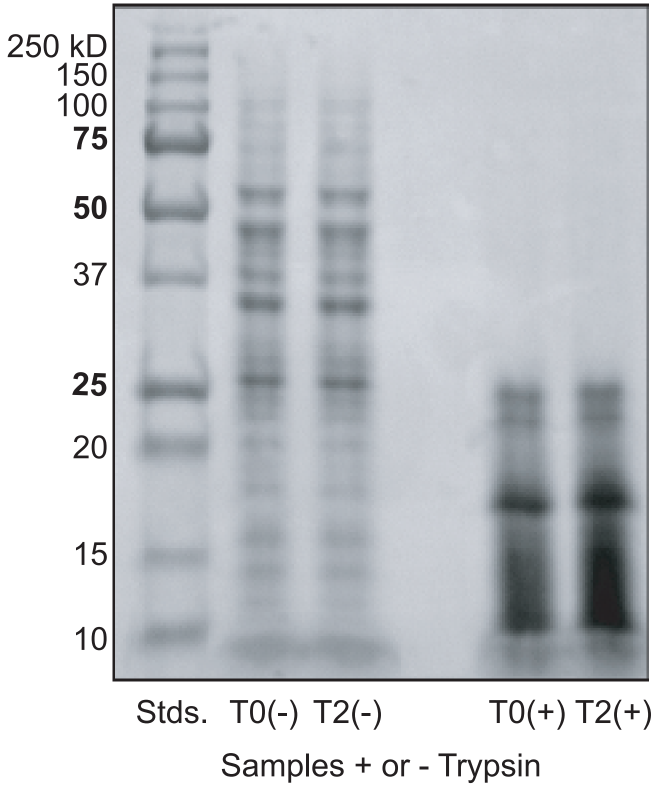
250 kD
150
100
75
50
37
25
20
15
10
Stds.
T0(-)
T2(-)
T0(+)
T2(+)
Samples + or - Trypsin

Samples Stationary Phase (SP) or Exponential (EX)
+ or – Trypsin (T), or Qiagen Protease (P), or Proteinase K (K)

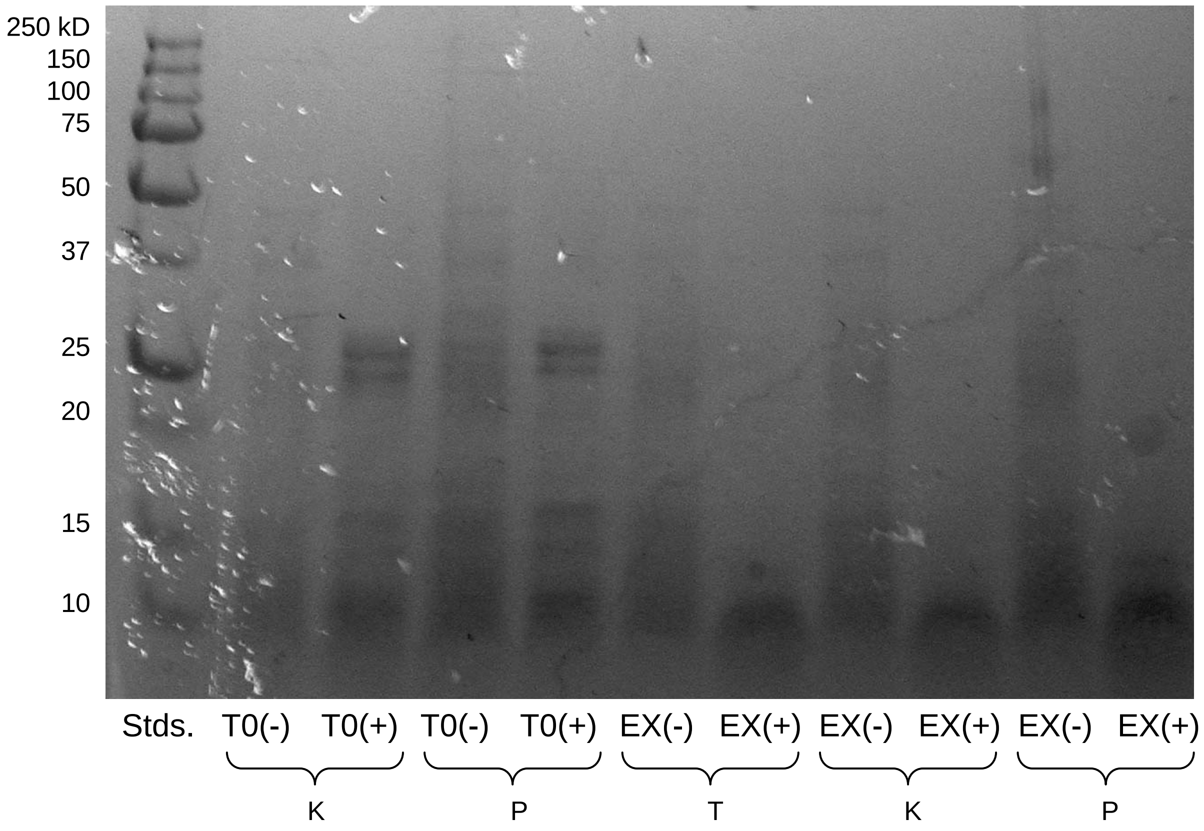

Supplement: Additional data file 4 — SDS-PAGE images. [file gb-2006-7-2-r9-S4.pdf]
