# Supplementary material for: Release of extraction-resistant mRNA in stationary phase Saccharomyces cerevisiae produces a massive increase in transcript abundance in response to stress
Source: Genome Biol. 2006 Feb 8;7(2):R9. doi: 10.1186/gb-2006-7-2-r9 (PMC1431719; doi:10.1186/gb-2006-7-2-r9)
Supplement: Additional data file 6 — Graph of quantitative RT-PCR. [file gb-2006-7-2-r9-S6.pdf]

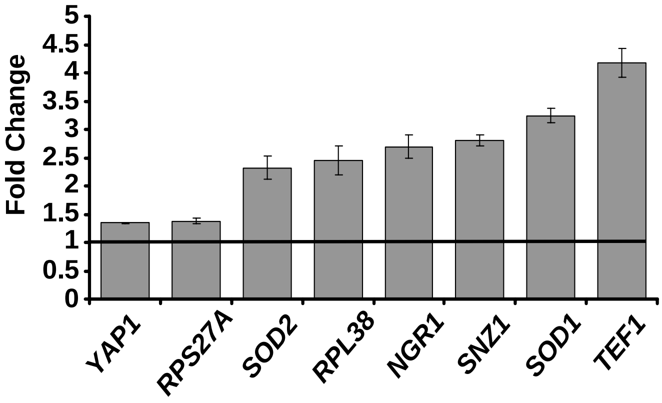

Fold change in the abundance of 8 transcripts between trypsin-treated extracts and extracts obtained with hot phenol. Fold change represents the difference in abundance between samples treated with trypsin during total RNA extraction versus total RNA extracted using hot phenol. Measurements were obtained by quantitative RT-PCR. Error bars represent the standard deviation of 3 measurements. The dark, horizontal bar at Fold Change = 1 indicates no change in transcript abundance. Values greater than 1 indicate a higher abundance of mRNA in the trypsin treated samples.
