# Supplementary material for: Release of extraction-resistant mRNA in stationary phase Saccharomyces cerevisiae produces a massive increase in transcript abundance in response to stress
Source: Genome Biol. 2006 Feb 8;7(2):R9. doi: 10.1186/gb-2006-7-2-r9 (PMC1431719; doi:10.1186/gb-2006-7-2-r9)
Supplement: Additional data file 8 — Detailed description of the RNA isolation protocol. [file gb-2006-7-2-r9-S8.pdf]

RNA Isolation Protocol

Cell Lysis

1. Collect 15-20 ODU (Optical Density Units) for Exponential Phase cells and 30-40 ODU for Stationary Phase and Post-Diauxic Shift cells per sample. Store pellets at –70C.

2. Add one scoopful of baked glass beads (0.5mm) using a homemade scoop with a PCR tube to the frozen cell pellet. (Rinse scoop w/ 95% EtOH before use.)

3. To frozen pellet add 300ul of room temp. **Cell Lysis Solution** (Gentra)

4. Use the bead beater in chromatography refrigerator (4ºC) (speed: 50 X 100 rpm) to lyse cells for 30 sec alternating with at least 30 sec on ice (Do this 6 times).

5. Centrifuge for 3 min in refrigerated centrifuge. Transfer supernatant to prechilled, new eppendorf tube. (All spins are @ max speed of 13,000 rpm)

Protease treatment (optional)

1. Split supernatant into equal halves 150 ul

2. To one half add appropriate amount of protease. To the other half add buffer alone.

3. Leave tube on ice for 1 hour inverting the tubes 20 times every 10 minutes

RNA precipitation:

1. Add 100ul of room temp **Protein-DNA Precipitation Solution** (Gentra) to supernatant.

2. Invert 10 times and put on ice for 5 min.

3. Centrifuge 3 min (refrigerated centrifuge).

4. Add supernatant to pre-chilled eppendorf tube containing 300ul of cold 100% isopropanol.

5. Mix by inversion at least 50 times, mix well

6. Centrifuge 3 min then remove supernatant. Note size and color of pellet.

7. Resuspend pellet in 100ul of DEPC-H_2O.

CH_3Cl: Phenol Treatment

1. Add 100ul of phenol:chloroform (5:1, **pH 4.5** from Ambion) to RNA.

2. Vortex 1 min, then centrifuge 5 min for a tight interface

3. Remove 80ul of aqueous phase to fresh eppendorf tube, avoid the interface and organic phase.

4. Back-extract = Add 80ul of depc-H_20 to phenol:chloroform and repeat steps 2 and 3.

5. Combine aqueous phases from steps 3 and 4.

6. EtOH precipitate the RNA by adding:
   0.1 times volume of 5M NH_4OAc
   2.5 times volume 100% EtOH

7. Precipitate overnight at –20°C, or if time is needed 15-30 min at –70°C.

8. Centrifuge precipitated RNA 20 min in refrigerated centrifuge.

9. Remove supernatant and wash RNA pellet with 0.5 ml of 70% RNase free EtOH. Vortex 1 min or until pellet is loosened from bottom.

10. Centrifuge 10min and remove EtOH.

11. Centrifuge additional 1min and remove residual EtOH.

12. Dry pellet 10-15min in air (place a lint-free wipe in a tip box, then open Epindorf tube caps and turn the tubes upside down on the lint-free wipe).

13. Resuspend in 50ul DEPC-water. Store at –70°C if not going on to next step immediately.

<u>Quantitation</u> (*Pre-Rneasy Quantitation)*

Quantitate all RNAs with the spectrophotometer. Dilute RNA in 10mM Tris-HCl pH 7.5 to so that peak absorbance at 260 nm is <1.2 OD. (Typically 1:50, dilution factor (DF)= 50).

<u>Rneasy (Qiagen) Cleanup</u> (The Rneasy column can hold a maximum of 100 ug.)

1. Adjust sample to a volume of 100 µl with RNase-free water.

2. Add 350 µl Buffer RLT and mix thoroughly.

3. Add 250 µl ethanol (100%) to the diluted RNA, and mix thoroughly by pipetting.
Do not centrifuge. Continue immediately with step 3.

4. Apply the sample (700 µl) to an RNeasy mini column placed in a 2 ml collection tube (supplied). Close the tube gently, and centrifuge for 15 sec at 8000 x *g* ≤10,000 rpm (usually do 13,000 rpm). Discard the flow-through and collection tube.

<u>DNaseI Treatment</u> *(steps 1-8 correspond to steps D1-D8 from the Rnase-Free-DNase Set (Qiagen) Protocol (1/1999)*
Prepare DnaseI stock according to the manufacturer's protocol

1. Pipette 350 ul Buffer RW1 into the spin column, and centrifuge for 15 sec at _8000 x g to wash. Discard flow –through and reuse collection tube.

2. Add 10 µl DNase I stock solution to 70µl Buffer RDD. Mix by gently inverting the tube.

3. Pipette the DNase I incubation mix (80µl) directly onto the spin-column membrane and place on the bench top for 15 minutes.

4. Pipette 350µl Buffer RW1 into the spin column and centrifuge for 15 seconds at ≤ 8000 rpm (usually 13,000 rpm). Discard flow-through.

5. Place the spin column in a new 2-ml collection tube. Pipette 500µl of Buffer RPE into the spin column and centrifuge for 15 seconds at ≤ 8000 rpm (usually 13,000 rpm). Discard flow-through.

6. Pipette 500µl Buffer RPE into the spin column and centrifuge for 2 minutes at maximum speed. Discard flow-through.

7. Spin 30 seconds to dry the silica-gel membrane. Remove column from collection tube. Make sure the column does not touch the ethanol flow-through!

8. Transfer the column to a new 1.5 ml collection tube and pipette 30ul of depc-H_20 directly onto the silica-gel membrane. Centrifuge for 1 minute at $\leq$ 8000 rpm (usually 13,000 rpm).

9. Repeat step 8 if the expected RNA yield is >30µg (this 2nd elution is typically performed).

10. Store RNA at -70°C.

Quantitation (*Post-Rneasy Quantitation)*

Quantitate all RNAs with the spectrophotometer. Dilute RNA in 10mM Tris-HCl pH 7.5 to so that peak absorbance at 260 nm is <1.2 OD. (Typically 1:50, dilution factor (DF) = 50).
