# Supplementary material for: Release of extraction-resistant mRNA in stationary phase Saccharomyces cerevisiae produces a massive increase in transcript abundance in response to stress
Source: Genome Biol. 2006 Feb 8;7(2):R9. doi: 10.1186/gb-2006-7-2-r9 (PMC1431719; doi:10.1186/gb-2006-7-2-r9)
Supplement: Additional data file 9 — Detailed description of the labeling protocol. [file gb-2006-7-2-r9-S9.pdf]

# Labeling Protocol

## A. Synthesis of cyanine-labeled cDNA

1. Program a thermocycler as follows:

**Program CMT**

| **Temperature** | **Time** | **Reaction** |
|---|---|---|
| 70°C | 10 min | Denaturation of template RNA |
| 4°C | 5 min | **Can pause thermocycler at this point** |
| 23°C | 10 min | Annealing of primer to RNA |
| 42°C | 2 h | Extension of cDNA chain |
| 4°C | hold | |

2. Depending on the RNA template, prepare the appropriate RNA/primer annealing mix on ice:

| **For Total RNA Templates** | | **For mRNA Templates** | |
|---|---|---|---|
| Total RNA | 20 µg | mRNA | 1.5 µg |
| Oligo(dT) primer | 2 µl (stock 1µ/µl) | Random primers (9-mers) | 3.3 µg |
| *A. thaliana* spikes | 1µl | *A. thaliana* spikes | 1µl |
| Nuclease-free water to: | 23 µl (total vol.) | Nuclease-free water to: | 23 µl |

**3.** Place tubes in the thermocycler and initiate the cycling program. **The *A. thaliana* spikes should be at a final concentration of 0.25ng/µl per reaction for each spike.**

4. Perform this step and the next during incubation of the RNA/primer mix. Prepare working stock of dNTPs having the following concentrations: 0.5 mM dATP, dGTP, dTTP, and 0.25 mM dCTP in DEPC-$H_2O$.
5. Keeping all reagents on ice, add in the following order (if performing multiple cDNA synthesis reactions, a single labeling mix for each dye can be prepared by scaling up the volumes proportionally):

| **Labeling Master Mix:** | **Cy3™ labeling** |
|---|---|
| 5 X first-strand buffer | 8 µl |
| 0.1 M DTT | 4 µl |
| dNTPs (from working stock prepared in step A4) | 2 µl |
| | **14µl** |
| 1 mM FluoroLink™ Cy3™-dCTP or Cy5™-dCTP | 1 µl |
| SuperScript™ II Reverse Transcriptase (200 U/µl) | 2 µl |

6. Initiate the cDNA synthesis reaction by adding in the following order: the labeling mix, dye, and SSII RT from step A5 to the RNA/primer mix of step A3, during the initial 5 minute hold at 4°C. The final reaction volume of the reaction is **40 µl**. Continue the thermocyler program (10 min at 23ºC followed by 2 h at 42ºC).

7. To the labeling reaction from step A6 add 1 µl of RNase H (2 U/µl) and 0.5 µl of RNase A (3.2 U/µl). Incubate at 37°C for 15 min.

## B. Probe Purification

1. Transfer labeling reactions to 1.5ml E-tubes to perform the precipitation and cleanup.
2. To precipitate the labeled cDNA, add 4 µl of 3 M sodium acetate, pH 5.2, to the 41.5-µl cDNA-synthesis reaction from step B2, mix briefly, then add 100 µl of 100% ethanol. Briefly mix the contents of the tube and incubate at –20ºC for at least 30 min. The cDNA may be stored at –20ºC for

up to one week. This is a good stopping point if the purification procedure cannot be completed within the day. Following incubation at –20ºC, centrifuge at 12,000 x g for at least 30 min to pellet the cDNA.

3. Carefully remove the supernatant and dissolve the pellet in 40 µl of DEPC treated water. Add 4 µl of 3 M sodium acetate, pH 5.2, and 200 µl of QIAquick™ Buffer PB. Incubate at 37ºC for 15 min (incubation at 95ºC for 30 sec is sometimes necessary to fully dissolve the pelleted cDNA).
4. Place a QIAquick™ column in a 2-ml collection tube. If combining several labeling reactions, keep in mind that these columns have a binding capacity of approximately 10 µg. For the purpose of purification, assume a yield of 1 µg of cDNA per reaction. Apply the cDNA/Buffer PB mixture to the center of the column.
5. Centrifuge column at 14,000 rpm for 60 sec in a microcentrifuge.
6. Discard flow-through and place column back in the collection tube.
7. Add 600 µl of QIAquick™ Buffer PE to the column. Centrifuge at 14,000 rpm for 1 min or until buffer has passed through column.
8. Discard flow-through and place column back in the collection tube.
9. Repeat steps C6 and C7 once more for a total of two 600-µl washes with Buffer PE.
10. Centrifuge the column for 2 min at 14,000 rpm to dry off the ethanol.
11. Place the column in a clean 1.5-ml microfuge tube. Apply 35 µl of elution buffer (we recommend the use of distilled water with pH adjusted to 8.0 by addition of a small volume of NaOH**)** preheated to 37ºC to the center of the resin bed and let stand for 1 min.
12. Centrifuge column at 14,000 rpm for 1 min.
13. Repeat the elution step with a second 35 µl of elution buffer (see C10). Optionally, DTT can be added to the eluate at a final concentration of 1 mM, to stabilize the cDNA.

Note: The QIAquick™ PCR columns eliminate single-stranded DNA molecule less than 200 nucleotides long (100 bp for double-stranded DNA). Failure to remove these small fragments from the probe may result in higher backgrounds.

## C. Determination of the Frequency of Incorporation of Fluorescent Nucleotide into the Probe

The frequency of incorporation (FOI) is defined as the number of labeled nucleotides incorporated per 1,000 nucleotides of cDNA. Use the following formulae to calculate the FOI:

**Amount of cDNA (ng) =** $A_{260}$ x 37 x total volume of probe (µl) **(Usually 70µl)**

**Total pmol of dye incorporated =**

for Cy3™-cDNA: $A_{550}$ x total volume of probe / 0.15
for Cy5™-cDNA: $A_{650}$ x total volume of probe / 0.25

**FOI =** pmol of dye incorporated x 324.5 / ng of cDNA

Note: Measurements should be done using undiluted probe directly in an ultra microcuvette. The probe should not be diluted for spectrophotometry because of its low initial concentration. Diluting the probe prior to assay may give inaccurate readings because of the low absorbance. The probe used for spectrophotometry should be recovered from the cuvette and used in the hybridization reaction. Clean the cuvette thoroughly between samples with 0.1 N HCl and nuclease-free water to prevent cross contamination.
