# Supplementary material for: Release of extraction-resistant mRNA in stationary phase Saccharomyces cerevisiae produces a massive increase in transcript abundance in response to stress
Source: Genome Biol. 2006 Feb 8;7(2):R9. doi: 10.1186/gb-2006-7-2-r9 (PMC1431719; doi:10.1186/gb-2006-7-2-r9)
Supplement: Additional data file 10 — Detailed description of the hybridization protocol. [file gb-2006-7-2-r9-S10.pdf]

HYBRIDIZATION

1. Pre-scan all of your chosen slides at 600 PMTfor both channels and 10 µm resolution. Visually check for the presence of background and make a decision about the quality, depending on your own standards. 50-80% of the green background speckles will be removed in the prehyb.

2. Prepare solutions.
The following solutions may form precipitates if chilled, which may fluoresce and ruin your data.

Prehybridization solution

	**80ml** per coplin jar	Recipe for **200ml**	Recipe for **600ml**
50% Formamide	40 ml stock	100ml stock	300ml stock
5 x SSC	20 ml of 20X stock	50 ml 20X stock	150ml 20X stock
0.1% SDS	800 µl of 10%	2ml 10% SDS	6ml 10% SDS
0.1 mg/ml BSA	800 µl of 10 mg/ml	2ml 10 mg/ml stock	6ml 10mg/ml stock
ddH_2O	18.4 ml ddH_2O	46ml dd-H_2O	138ml dd-H_2O

Add Formamide, water, SDS and SSC, filter sterilize with 0.2 µm Nylon filter (big filter apparatus attached to 250ml bottle), **then add BSA**

Hybridization solution

	**Make 10 ml Premix** (make in a 15ml Falcon tube)
50% Formamide	5 ml stock
5% Dextran sulfate	0.5 g
5X SSC	2.5 ml (20X stock)
0.1% SDS	100 µl (10% SDS stock)
0.1 mg/ml BSA	100 µl (10 mg/ml stock)
ddH_20	2.2 ml ddH_20
100 µg/ml Salmon sperm DNA	-----

Procedure:
Add Dextran sulfate to water and dissolve at 37-42ºC. Add formamide, SSC, and SDS. Filter sterilize with 0.2 µm Nylon filter (can use luer-lock syringe with a filter), add BSA, then aliquot (see below).

Hybridization solution **(enough for approx. 8 slides)**
297 µl premix
3 µl Salmon Sperm DNA (10 mg/ml stock)

3. Put prehybridization solution in a glass coplin jar in the 42ºC water bath and let sit at least ½ hour before proceeding to the next step.

4. A. Prehybridize slides in appropriate volume of Prehybridization solution in a Coplin Jar at 42°C for 1-2 hours. Rinse the slide briefly (5 sec) with ddH_20 from a squirt bottle and then dip in 100% ethanol for 1 second. Dry under 15-30psi N_2 gas stream to push off all droplets of liquid. Do not allow liquid to dry on the slide.

B. Clean the cover slips by dipping in a 1M KOH and 50% EtOH solution, then squirting with ddH_2O, and drying with 30psi of N_2. (this can be done while the slides are undergoing prehybridization. Keep in dust free box until use). Do not let pre-hybed slides sit dry for more than about ½ hour before hybing.

C. Turn the heat block on to 95ºC.

5. Prepare labeled target for the hybridization
Add both the Cy3 and Cy5 reactions used for one slide to the same tube, then dry them down together. Dry the labeled targets in speed vac (lyophilizer) with heat for about 1-3 hours, or until dry. **Do not over dry**. Resuspend in hybridization buffer.
Heat mixture to 95°C for 5 minutes to denature DNA, then spin for 30 sec.

<u>Our suggested volumes for coverslips:</u>

24X30 LifterSlip…………….**.35 μl**

6. Begin hybridization – work without haste or interruption

Pipette labeled target onto the underside of the LifterSlip into one large droplet, which is set on a flat clean surface (avoid dispensing bubbles), then place the slide onto the LifterSlip, turn the slide right-side up.

Enclose the slide in a CMT chamber. Add **15μl** of water to each hydration well. Tape the closed chambers to the orbital platform or rocker in an oven set to 42°C and incubate for 16 to 20 hours.

WASHING

Wash buffers should be filter sterilized to remove dust. Reduce light exposure to slides whenever possible. The first wash solution is typically left in 42ºC water bath without shaking if it has precipitated. The LifterSlip should come off during this wash, if not, gently rock by hand until it does. Do not allow the coverslip to scratch the slide surface.

Place slides in a glass Coplin jar (large enough to hold all of the slides at once) on the rocker, cover with foil to keep light out.

After the last wash, dry under 15-30 psi stream of N_2 gas. Place back in clean chamber or slide holder and scan.

Wash Buffer (Temp.)	Wash Time	How	Number of Washes
2 x SSC, 0.1% SDS (42ºC)	1 min	in 42ºC bath	1
2 x SSC, 0.1% SDS (42ºC)	5 min	on rocker	1
0.1 x SSC, 0.1% SDS (RT)	10 min	on rocker	1
0.1 x SSC (RT)	10-20 sec	dip	1
0.1 x SSC (RT)	2 min	on rocker	2
0.1 x SSC (RT)	1 min	on rocker	1
0.01 x SSC (RT)	15 sec	dip	1

To make 1 liter of each wash solution:

2 x SSC, 0.1% SDS

100ml	20 x SSC
10ml	10% SDS
890ml	ddH_2O

0.1 x SSC, 0.1% SDS

5ml	20 x SSC
10ml	10% SDS
985ml	ddH_2O

0.1 x SSC

5ml	20 x SSC
995ml	ddH_2O

0.01 x SSC

0.5ml	20 x SSC
999.5ml	ddH_2O
