# Supplementary material for: Release of extraction-resistant mRNA in stationary phase Saccharomyces cerevisiae produces a massive increase in transcript abundance in response to stress
Source: Genome Biol. 2006 Feb 8;7(2):R9. doi: 10.1186/gb-2006-7-2-r9 (PMC1431719; doi:10.1186/gb-2006-7-2-r9)
Supplement: Additional data file 11 — Detailed description of the ANOVA measurement model. [file gb-2006-7-2-r9-S11.pdf]

Correlation Between Biological and Technical Replicates. To evaluate reproducibility of our experimental technique as well as between T_0 samples, a statistical measurement model was generated from microarray data from six independent T_0 samples. The measurement model represents the observed $\log_2$ (R/G) values in terms of specific effects due to gene, culture, and replicate. Let Y_{ijk} denote the measured $\log_2$ (R/G) value of the ith gene for the kth replicate slide within the jth culture. The model is given by $Y_{ijk} = \mu_i + \alpha_j + \beta_{k(j)} + \varepsilon_{ijk}$, where μ_i represents the underlying true/idealized (but unknown) value of the log-ratio for the ith gene, α_j denotes a global effect (across all genes) that is present during the measurement of slides from the jth culture, $\beta_{k(j)}$ denotes an effect on the measurement (across all genes) associated with the kth replicate slide within the jth culture, and ε_{ijk} represents the specific effect of the kth replicate slide within the jth culture on the ith gene. The ith gene is said to be up-regulated or down-regulated (treatment versus control) if the estimate of μ_i ($\hat{\mu}_i$) is significantly different than zero. Examination of μ_i shows data between 1 and -1 $\log_2$ demonstrating no significant differences between the T_0 samples (Fig. 1A).

The other effects (α_j, $\beta_{k(j)}$, and ε_{ijk}) are regarded as random measurement errors and thus hinder the ability to determine whether a gene is up- or down-regulated. In this case, the α_j effects are relatively large ($\hat{\sigma}_\alpha$ = 0.57) and are possibly a consequence of unique PMT settings for each culture. The $\beta_{k(j)}$ effects are smaller ($\hat{\sigma}_\beta$ = 0.08). In combination, the α_j and $\beta_{k(j)}$ effects introduce a specific global shift (across all genes) in log-ratios for each slide when comparing T_0 measurements averaged from culture two versus averaged from culture one (Fig. 1B). Presumably, these global effects can be effectively removed via

proper normalization. The other measurement error (ε_{ijk}) is gene-specific and much smaller than the α_j and $\beta_{k(j)}$ effects. A LOWESS smooth [54] of the raw standard deviations suggests that $\hat{\sigma}_\varepsilon$ is about 0.30 when $|\hat{\mu}| \geq 1$ (Fig. 1C). Thus, disregarding the α_j and $\beta_{k(j)}$ effects, any change greater than 1.6 fold can be viewed as statistically significant with a Type I error (or false positive rate) of about 0.01.

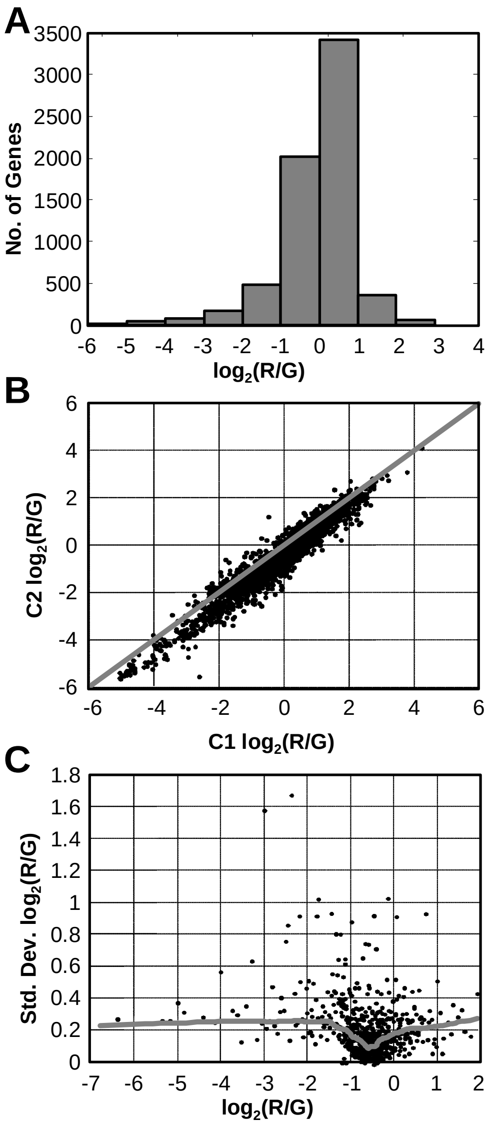

Fig 1. (*A*) Histogram of estimated values of μ $\log_2$ ratio. (*B*) Mean $\log_2$ ratio of culture 2 (C2) slides versus culture 1 (C1) slides (replicate T_0 samples). (*C*) Standard deviation of the ε_{ijk}'s as a function of $\hat{\mu}_i$ across the various genes with LOWESS smooth.

References

54. Cleveland WS: **Robust Locally Weighted Regression and Smoothing Scatterplots**. *Journal of the American Statistical Association* 1979, **74**(368):829-836.
