# Supplementary material for: Release of extraction-resistant mRNA in stationary phase Saccharomyces cerevisiae produces a massive increase in transcript abundance in response to stress
Source: Genome Biol. 2006 Feb 8;7(2):R9. doi: 10.1186/gb-2006-7-2-r9 (PMC1431719; doi:10.1186/gb-2006-7-2-r9)
Supplement: Additional data file 12 — Detailed description of the quantitative RT-PCR protocol. [file gb-2006-7-2-r9-S12.pdf]

RT-PCR Protocol

RT

Components for cDNA synthesis	Final volume 8.7 µL	3X RXN. Final volume 26.1 µL	Final amount
RNA (250ng/ul)	2 µL of dilution	6 µL of dilution	500 ng
Exogenous RNA Control (Cab)	1 µL (10 ng/µl)	1 µL (10 ng/µl)	10 ng
Random Hexamers (50µM)	1 µL	3 µL	2.5 µM
RNase free water	4.7 µL	16.1 µL	-----

Run the above samples on the **Thermocycler** using program **CMT**

	**Temperature**	**Time**	**Reaction**
**CMT:**	70°C	10 minutes	Denaturation of template RNA
	4°C	5 minutes	**PAUSE: Add Master Mix!!!!!**
	23°C	10 minutes	Annealing of primer to RNA
	42°C	2 hour	Extension of cDNA chain
	4°C	hold	

Final Components	Final volume 20 µL	Master Mix = 10 rxns.	Master Mix = 40 rxns.	Final concentration
10X TaqMan RT Buffer	2 µL	20 µL	80 µL	1X
25 mM $MgCl_2$	4.4 µL	44 µL	176 µL	5.5 mM
deoxyNTPs Mixture (2.5 mM)	4 µL	40 µL	160 µL	500 µM per dNTP
RNase Inhibitor (20 U/L)	0.4 µL	4 µL	16 µL	0.4 U/µL
MultiScribe Reverse Transcriptase (50 U/µL)	0.5 µL	5 µL	20 µL	1.25 U/µL

***Add 11.3 µL of Master Mix to each reaction at the 4°C step.**
***For 3X RXN. add 33.9 µL of Master Mix to each reaction at the 4°C step.**
Read the amount of cDNA (nucleic acid / W.C.) on the spectrophotometer, once program "CMT" is completed.

(1:50) dilution: (2µL of cDNA + 98µL 10mM Tris HCl pH 7.5)

The Applied Biosciences protocol for SybrGreen RT-PCR suggests 1-10ng of cDNA template and 3 μL of 5μM [] primers be used per reaction.

I typically make three times the following amounts in one tube, and then aliquot into three wells for triplicate reactions.

PCR

**Components**	**For 25 μL total vol.**	**No Template 25 μL**
2X SYBR green PCR master mix	12.5 μL	12.5 μL
Forward & Reverse Primers together (5μM each)	3.0 μL	3.0 μL
cDNA template (1-10 ng/μL)	1 μL	**None**
DEPC-H_2O	8.5 μL	9.5 μL

For multiple reactions:

**Components**	**For 100 μL total vol. (x 4 wells)**	**For 250 μL total vol. (x 10 wells)**	**For 750 μL total vol. (x 30 wells)**
2X SYBR green PCR master mix	50 μL	125 μL	375 μL
Forward & Reverse Primers together (5μM each)	12 μL	30 μL	90 μL
cDNA template (1-10 ng/μL)	2 μL	10 μL	30 μL
DEPC-H_2O	36 μL	85 μL	255 μL
